# Hazara Nairovirus Requires COPI Components in both Arf1-Dependent and Arf1-Independent Stages of Its Replication Cycle

**DOI:** 10.1128/JVI.00766-20

**Published:** 2020-08-17

**Authors:** J. Fuller, B. Álvarez-Rodríguez, E. J. A. A. Todd, J. Mankouri, R. Hewson, J. N. Barr

**Affiliations:** a School of Molecular and Cellular Biology, University of Leeds, Leeds, United Kingdom; b National Infection Service, Public Health England, Salisbury, United Kingdom; University of Kentucky College of Medicine

**Keywords:** COPI, cell biology, host factors, virology

## Abstract

Hazara nairovirus (HAZV) is an enveloped trisegmented negative-strand RNA virus classified within the *Nairoviridae* family of the *Bunyavirales* order and a member of the same subtype as Crimean-Congo hemorrhagic fever virus, responsible for fatal human disease. Nairoviral subversion of cellular trafficking pathways to permit viral entry, gene expression, assembly, and egress is poorly understood. Here, we generated a recombinant HAZV expressing enhanced green fluorescent protein and used live-cell fluorescent imaging to screen an siRNA library targeting genes involved in cellular trafficking networks, the first such screen for a nairovirus. The screen revealed prominent roles for subunits of the coat protein 1 (COPI)-vesicle coatomer, which regulates retrograde trafficking of cargo between the Golgi apparatus and the endoplasmic reticulum, as well as intra-Golgi transport. We show the requirement of COPI-coatomer subunits impacted at least two stages of the HAZV replication cycle: an early stage prior to and including gene expression and also a later stage during assembly and egress of infectious virus, with COPI-knockdown reducing titers by approximately 1,000-fold. Treatment of HAZV-infected cells with brefeldin A (BFA), an inhibitor of Arf1 activation required for COPI coatomer formation, revealed that this late COPI-dependent stage was Arf1 dependent, consistent with the established role of Arf1 in COPI vesicle formation. In contrast, the early COPI-dependent stage was Arf1 independent, with neither BFA treatment nor siRNA-mediated ARF1 knockdown affecting HAZV gene expression. HAZV exploitation of COPI components in a noncanonical Arf1-independent process suggests that COPI coatomer components may perform roles unrelated to vesicle formation, adding further complexity to our understanding of cargo-mediated transport.

**IMPORTANCE** Nairoviruses are tick-borne enveloped RNA viruses that include several pathogens responsible for fatal disease in humans and animals. Here, we analyzed host genes involved in trafficking networks to examine their involvement in nairovirus replication. We revealed important roles for genes that express multiple components of the COPI complex, which regulates transport of Golgi apparatus-resident cargos. COPI components influenced at least two stages of the nairovirus replication cycle: an early stage prior to and including gene expression and also a later stage during assembly of infectious virus, with COPI knockdown reducing titers by approximately 1,000-fold. Importantly, while the late stage was Arf1 dependent, as expected for canonical COPI vesicle formation, the early stage was found to be Arf1 independent, suggestive of a previously unreported function of COPI unrelated to vesicle formation. Collectively, these data improve our understanding of nairovirus host-pathogen interactions and suggest a new Arf1-independent role for components of the COPI coatomer complex.

## Abstract (precis)

Nairoviruses are tick-borne enveloped RNA viruses that include several pathogens responsible for fatal disease in humans and animals. Here, we analyzed host genes involved in trafficking networks to examine their involvement in nairovirus replication. We revealed important roles for genes that express multiple components of the COPI complex, which regulates transport of Golgi apparatus-resident cargos. COPI components influenced at least two stages of the nairovirus replication cycle: an early stage prior to and including gene expression and also a later stage during assembly of infectious virus, with COPI knockdown reducing titers by approximately 1,000-fold. Importantly, while the late stage was Arf1 dependent, as expected for canonical COPI vesicle formation, the early stage was found to be Arf1 independent, suggestive of a previously unreported function of COPI unrelated to vesicle formation. Collectively, these data improve our understanding of nairovirus host-pathogen interactions and suggest a new Arf1-independent role for components of the COPI coatomer complex.

## INTRODUCTION

The *Bunyavirales* order of enveloped, segmented negative-sense RNA viruses comprises a diverse collection of over 500 viruses that are classified into 12 families, with five of these containing the causative agents of human disease, namely, the *Hantaviridae*, *Nairoviridae*, *Arenaviridae*, *Peribunyaviridae*, and *Phenuiviridae* families (1). The nairoviruses currently comprise 12 named species, all of which are arboviruses, associated with hard ticks of the *Ixodidae* family, for which transmission to mammalian and avian hosts occurs through acquisition of a blood meal. The family was named after Nairobi sheep disease virus, which causes fever, hemorrhagic gastroenteritis, and abortion in goats and sheep and carries a case-fatality rate of around 80% resulting in considerable economic impact (2). Notable nairovirus member, priority pathogen Crimean-Congo hemorrhagic fever virus (CCHFV) is the responsible agent for Crimean-Congo hemorrhagic fever (CCHF), a devastating human disease with fatality rates averaging approximately 30% (3–5) for which preventative or therapeutic measures are not available. Concern surrounding the spread of CCHFV to new geographic locations is rising, due in major part to the altered habitat of the tick host, possibly in response to climate change, exemplified by recent fatal human infections in northern Spain (6, 7). Due to these factors, CCHFV is classified as an emerging hazard group 4 pathogen, and therefore research on this virus requires highly specialized laboratory facilities. CCHFV shares the same CCHFV serogroup with Hazara virus (HAZV), also possessing extensive similarities in the sequence, structure, and function of its constituent proteins (8–10). However, HAZV is not recognized as a serious human pathogen and is categorized as a hazard level 2 virus, facilitating its use as a model system for CCHFV in a containment level 2 laboratory infrastructure.

All nairoviruses possess a negative-stranded RNA genome composed of three segments named small (S), medium (M), and large (L) based on their relative sizes. Each genome template is transcribed to yield a single mRNA; the S mRNA encodes the RNA-binding nucleocapsid protein (N); the M segment mRNA encodes a glycoprotein precursor that is cleaved into envelope spikes Gn and Gc, as well as nonstructural NSm; and the L segment mRNA encodes the large RNA-dependent RNA polymerase responsible for all viral RNA synthesis activities. An additional nonstructural protein NSs has been described expressed by ambisense transcription from the CCHFV S segment antigenome, with possible roles in apoptotic signaling (11). Whether HAZV expresses an analogous product is unknown.

Despite the importance of nairoviruses due to their impact on both human and animal health, knowledge of the nairovirus replication cycle at the molecular level is still lacking. Nairovirus particles infect cells through a clathrin- and Rab5-dependent process (12–14), enhanced by DC-SIGN (15), and the RNA segments escape the endocytic system from multivesicular bodies (MVBs) (13), mediated by the Gn/Gc spikes that orchestrate fusion of the MVB and viral envelopes in response to the resident biochemical milieu (16). Following release, the viral segments likely traffic in a microtubule-dependent manner (17) to a currently elusive cellular compartment to establish replication factories, within which gene expression, RNA replication, protein synthesis, and subsequent virus assembly occurs. By analogy with Bunyamwera virus (BUNV), the prototypical *Bunyavirales* order member, one candidate location for these factories is the Golgi apparatus; BUNV establishes its replication factories surrounding the Golgi stacks (18), inducing the formation of specialized viral tubes that accumulate viral components, with subsequent assembly and maturation of virions involving the passage through Golgi subcompartments prior to virus budding from secretory vesicles (19, 20). This utilization of cellular endomembranes to support various viral processes is a common strategy adopted by enveloped viruses but is poorly understood for nairoviruses.

A detailed picture of how nairoviruses exploit cellular trafficking networks during all stages of the virus replication cycle have been limited by the paucity of virus-associated tools that permit large screening approaches. In this study, we used our recently developed reverse genetics system for HAZV (21) to rescue a recombinant HAZV expressing enhanced green fluorescent protein (rHAZV-eGFP) as a nonfused reporter protein, by inserting a P2A ribosome-skipping sequence from porcine teschovirus (22–24) within the HAZV S segment mRNA, as has recently been reported for CCHFV (25). We assessed the trafficking components required for rHAZV-eGFP infection using a targeted library of small interfering RNAs (siRNAs) specific for cellular components involved in vesicular transport and measured their influence on virus growth by live cell fluorescence microscopy. We revealed a role for coat protein 1 (COPI)-vesicle coatomer subunits, which are primarily involved in retrograde trafficking of cargo between the Golgi apparatus and the endoplasmic reticulum (ER) and intra-Golgi transport, but with additional reported roles in maintaining functionality of the endocytic network (26–28), breakdown of the nuclear envelope (29), and lipid homeostasis (30–32). The requirement of COPI-coatomer subunits for HAZV growth impacted an early stage of the virus replication cycle, prior to translation, as well as a later stage, involving infectious virus assembly. While the late stage was Arf1 dependent, as expected for canonical COPI vesicle formation, the early stage was found to be Arf1 independent, suggestive of a previously unreported function of COPI unrelated to vesicle formation. These data provide new insight in our understanding of how nairoviruses usurp important cellular trafficking components to establish a productive infection.

## RESULTS

### Generation of a recombinant HAZV expressing enhanced green fluorescent protein.

**FIG 1:**
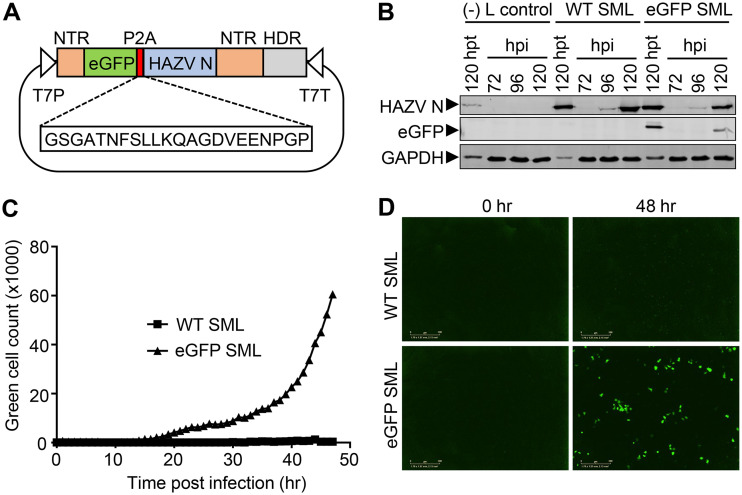
Rescue of rHAZV expressing eGFP. (A) Schematic representation of pMK-RQ-S(eGFP), encoding a modified HAZV S segment, designed to express eGFP and N using a porcine teschovirus-1 2A peptide linker (P2A). The sequence of the P2A region is shown, intervening the ORFs for eGFP, HAZV nucleoprotein (HAZV N), and flanked by nontranslated regions (NTR). The hepatitis delta virus ribozyme (HDR) and T7 RNA polymerase promoter and terminator signals (T7P/T7T) are also shown for reference. (B) BSR-T7 cells were transfected with either WT S, M, and L rescue plasmids (WT SML) or with plasmids required for rescue of rHAZV-eGFP (eGFP SML). At 120 hpt, supernatants were transferred to naive SW13 cells. Lysates from BSR-T7 at 120 hpt and SW13 cells at 72, 96, and 120 hpi were analyzed for expression of eGFP and HAZV N by Western blot analysis using the corresponding antisera. Lysates were also collected from a recovery experiment in which the L segment expression plasmid was omitted [(–)L control]. (C) Green cell count at hourly intervals during a 48-h time course infection of SW13 cells with either rHAZV-eGFP or WT rHAZV. (D) Representative live cell images taken under UV illumination at 0 and 48 hpi of SW13 cells.

To facilitate high-throughput siRNA screens to determine the involvement of cellular trafficking factors in HAZV replication, we generated a recombinant HAZV (rHAZV) expressing eGFP. The expression strategy adopted involved the porcine teschovirus-1 2A peptide linker (P2A) sequence that induces ribosome skipping (22–24), to allow two open reading frames (ORFs) to be expressed from the HAZV S segment mRNA. The P2A linker was inserted between eGFP and HAZV N ORFs within previously described S segment expression plasmid (pMK-RQ-S) (21) to generate pMK-RQ-S(eGFP) (Fig. 1A).

Transfection of modified plasmid pMK-RQ-S(eGFP), along with wild-type (WT) M and L segment expression plasmids, was performed alongside a transfection comprising all three WT plasmids for comparison. An additional plasmid, pCAGGS-T7pol, which expresses bacteriophage T7 RNA polymerase was also cotransfected into cells, due to its previously described beneficial effects on the recovery of rHAZV (21, 33). At 120 h posttransfection (hpt), supernatants were harvested and used to reinfect fresh monolayers of SW13s. Western blot analysis of corresponding lysates at 72, 96, and 120 h postinfection (hpi) revealed abundant HAZV N expression confirming successful rescue of both rHAZV and rHAZV-eGFP viruses (Fig. 1B). As expected, no HAZV N was detected in fresh cells following transfer of supernatants from control rescues in which the L segment expressing plasmid was omitted. As further confirmation of rHAZV-eGFP rescue, the eGFP signal was exclusively detected in rHAZV-eGFP-infected SW13 cell lysates following Western blot analysis (Fig. 1B).

The purpose of generating rHAZV-eGFP was to allow the rapid and quantitative measurement of HAZV gene expression through the detection of eGFP fluorescence using live cell imaging. To test the utility of this assay, SW13 cells were infected with supernatants containing either rHAZV or rHAZV-eGFP collected at 120 hpt, with infected cells analyzed using a live cell imaging IncuCyte system at hourly intervals. Quantification of the green cell count showed increasing numbers of eGFP-expressing infected cells over the 48-h duration confirming detectable levels of rHAZV-eGFP infection (Fig. 1C and D).

**FIG 2:**
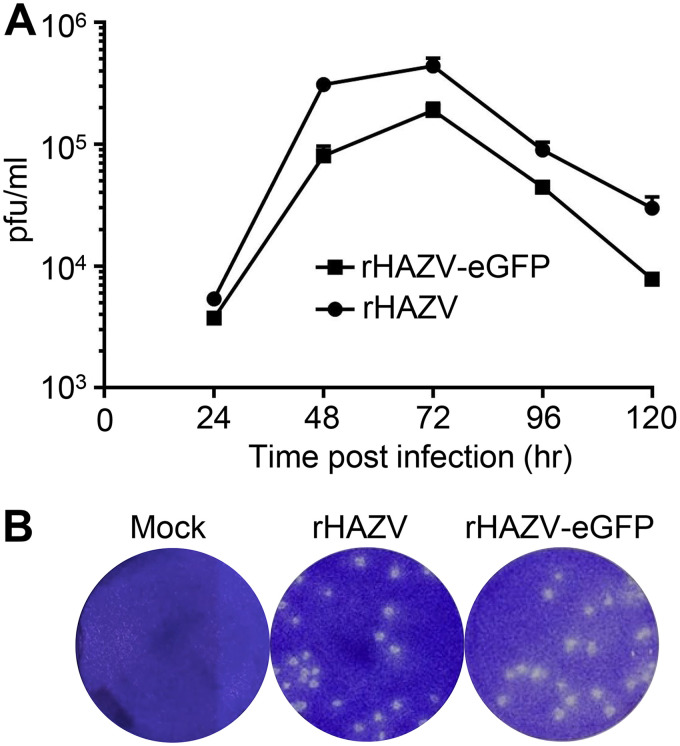
Growth comparison of rHAZV and rHAZV-eGFP. (A) Titers of infectious supernatant harvested at 24, 48, 72, and 96 hpi from SW13 cells infected with either recombinant Hazara virus (rHAZV) or rHAZV expressing eGFP (rHAZV-eGFP) at an MOI of 0.01. Error bars show the variance over three independent experiments. (B) Representative plaque assays from mock-, rHAZV-, and rHAZV-eGFP-infected SW13 cells at 6 days postinfection, showing indistinguishable plaque size and morphology.

We next compared the growth properties of rHAZV and rHAZV-eGFP to determine the impact of the addition of the eGFP ORF and P2A linker on overall virus fitness. Titers of stocks of both viruses were determined by plaque assay and used to infect fresh SW13 cells at a multiplicity of infection (MOI) of 0.01. Cell supernatants were collected at 24-h intervals across a 5-day period, and the titers of released viruses were further assessed by plaque assays. This analysis revealed that titers for WT rHAZV and rHAZV-eGFP were similar across the entire 5-day time period, with both viruses exhibiting peak titers at 72 hpi for which rHAZV titers were approximately 2-fold higher (Fig. 2A). The plaque size and morphology for rHAZV and rHAZV-eGFP remained indistinguishable (Fig. 2B). Taken together, these findings suggest that rHAZV and rHAZV-eGFP possess broadly similar growth parameters, demonstrating that rHAZV-eGFP represents a useful tool for studying nairovirus growth and infectivity.

### Identification of the trafficking components required during HAZV replication.

We next used rHAZV-eGFP to identify key host cell trafficking components that play a role during HAZV growth, achieved using a library comprising three unique siRNAs for each of 142 gene targets. Following transfection of each unique siRNA, SW13 cells were infected with rHAZV-eGFP at an MOI of 0.25 for 24 h, and the total integrated intensity of the eGFP (TIIE) signal was quantified as a marker of rHAZV-eGFP growth. At this 24-h time point, no cells newly infected and fluorescing cells are detected; thus, quantification of the eGFP signal recorded at 24 hpi would mostly reflect the influence of siRNA knockdown on virus replication cycle stages up to and including eGFP expression, within the first round of infected cells. The influence of knockdown on later events in the growth cycle, including virus assembly, budding, and infection of new cells, would not be represented in the TIIE signal. The effect of each unique siRNA was tested four times (see Data Set S1 in the supplemental material), and the 25 gene identities associated with greatest overall reduction in TIIE signal, over the mean of all three unique siRNA knockdowns against each gene target, are summarized in Table 1.

**TABLE 1:**
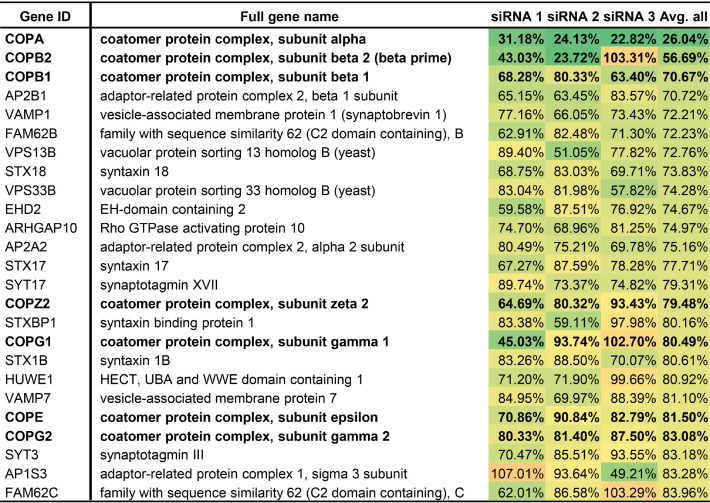
Listing of top 25 siRNA targets based on percentage knockdown of total integrated intensity of eGFP signal across three unique siRNAs^*a*^

a Each percentage value represents the mean of four experimental repeats, and colors indicate the levels of knockdown from green (most knockdown) to orange (least knockdown). Genes highlighted in boldface represent those associated with COPI vesicles.

### COPI components are important for HAZV intracellular growth.

**FIG 3:**
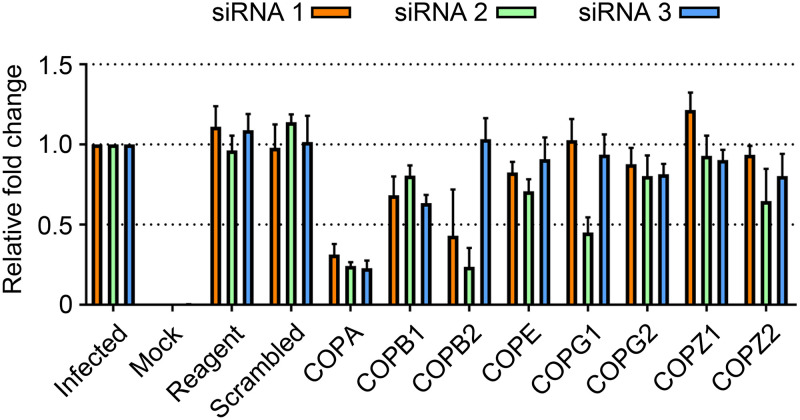
HAZV gene expression in first-round infected cells requires several components of the COPI complex. Histogram of the relative fold change in total integrated intensity of eGFP signal in SW13 cells infected with rHAZV-eGFP at an MOI of 0.25, following independent reverse transfection of triplicate siRNAs (siRNA 1, orange bars; siRNA 2, green bars; siRNA 3, blue bars) against the indicated cellular genes that express components of COPI complexes. Transfection reagent only and scrambled siRNA transfections were included as controls.

The cell factors for which siRNA knockdown resulted in greatest TIIE reduction (Table 1) include seven genes expressing COPI subunits, including components of both the cage-like (α, β′, and ε) and adapter-like (β, γ, δ, and ζ) subcomplexes (34, 35). The three most impactful knockdowns corresponded to COPA, COPB1, and COPB2 genes, encoding α, β, and β′ subunits, respectively (Fig. 3); siRNA knockdown of COPA had the largest effect on eGFP signal with all three unique siRNAs displaying 70 to 80% reduction compared to untreated controls (siRNA 1 = 0.311 [*P* = 0.0003]; siRNA 2 = 0.241 [*P* < 0.0001]; siRNA 3 = 0.228 [*P* = 0.0001]). COPB1 knockdown also caused a significant reduction of the eGFP signal with all three siRNAs, but to a lesser degree, displaying between 20 and 40% reduction versus untreated controls (siRNA 1 = 0.682 [*P* = 0.0127]; siRNA 2 = 0.803 [*P* = 0.0078]; siRNA 3 = 0.634 [*P* = 0.0008]). COPB2 knockdown reduced the eGFP signal significantly in two of three siRNAs (siRNA 1 = 0.430 [*P* = 0.0289]; siRNA 2 = 0.237 [*P* = 0.0009]; siRNA 3 = 1.033 [*P* = 0.6497]), as did COPE knockdown (siRNA 1 = 0.709 [*P* = 0.0043]; siRNA 2 = 0.908 [*P* = 0.2701]; siRNA 3 = 0.828 [*P* = 0.0146]). siRNA knockdown of other complex subunits COPZ2, COPG1, and COPG2 resulted in mean TIIE reductions of around 20%, but with lower overall significance. In contrast, COPZ1 knockdown had no influence on TIIE (Fig. 3).

### Validation of successful COPA, COPB2, and COPB1 silencing.

**FIG 4:**
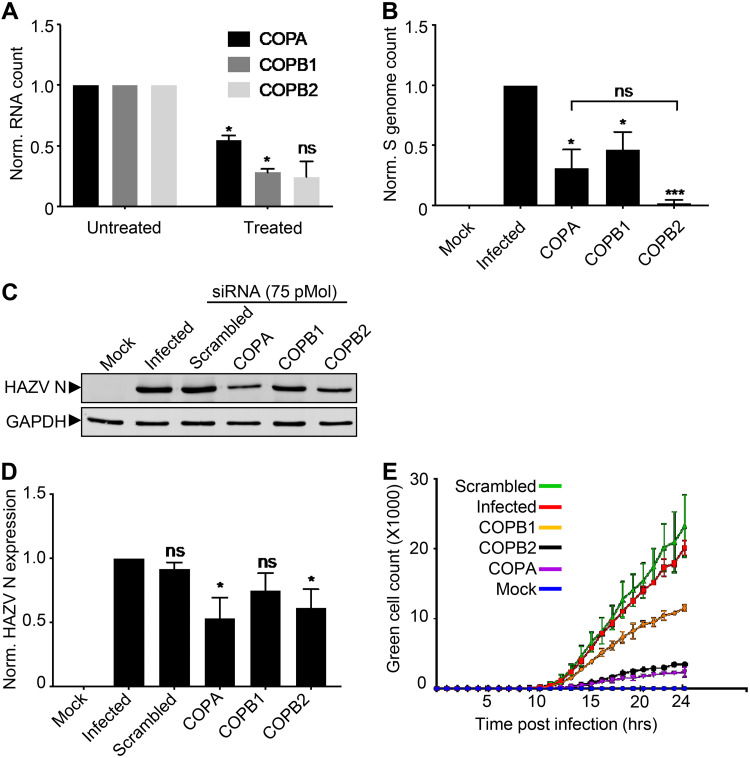
Validation of the effects of COPI complex knockdown on COPI expression, HAZV N protein expression, viral replication, and green cell count. (A) qRT-PCR analysis of COPI subunit gene expression in SW13 cells transfected with siRNAs targeting COPA, COPB1, and COPB2, with error bars representing data from two experimental repeats. (B) qRT-PCR analysis of HAZV S segment-specific RNAs in rHAZV-infected SW13 cell lysates after treatment with siRNAs targeting COPA, COPB1, and COPB2 at 24 hpi. Mock-infected (Mock) and infected (Infected) samples were added. (C) Western blot analysis using HAZV N antisera of rHAZV-infected SW13 cell lysates after treatment with siRNAs targeting COPA, COPB1, and COPB2. A scrambled control (Scrambled) was also included alongside a mock-infected (Mock) and infected (Infected) sample in which transfection reagent but no siRNA was added, with GAPDH detected by corresponding antisera, as a loading control. The resulting band intensities on blots from three independent experiments were quantified by densitometry, represented graphically in panel D. (E) Hourly measurement of eGFP expression in SW13 cells infected with rHAZV-eGFP following treatment with siRNAs targeting COPA, COPB1, and COPB2 over a 24-h time course. The statistical significance between conditions is indicated (ns, not significant; *, *P* < 0.1; **, *P* < 0.01; ***, *P* < 0.001).

For the three cellular genes with the greatest impact on rHAZV-eGFP expression, namely, COPI coat-like complex components COPA and COPB2 and the adapter-like component COPB1, further validation of their observed impact was performed using infections WT rHAZV. First, confirmation of the knockdown of the corresponding genes was achieved using quantitative reverse transcriptase PCR (qRT-PCR) targeting COPA, COPB1, and COPB2, for which mRNA copy numbers in knockdown cells were reduced in all three cases by ≥50% compared to the untreated infection controls (Fig. 4A). Next, we measured the effect of COPA, COPB1, and COPB2 knockdown on rHAZV RNA synthesis through quantifying the abundance of intracellular HAZV S segment copy numbers by qRT-PCR analysis. The abundance of S segment-specific RNAs were reduced by ≥50% in all three cases (Fig. 4B), and while the reduction in S segment RNA abundance following COPB2 knockdown is greater than that for COPA or COPB1 knockdowns, none of the observed differences are statistically significant. Next, we examined HAZV N protein abundance by Western blot analysis using N protein antisera, which confirmed reduced N protein levels following the silencing of all three COPI components (Fig. 4C and D), a finding in close agreement with the reduction in TIIE (Table 1 and Fig. 3). Finally, the expression of eGFP from rHAZV-eGFP infected cells following independent COPA, COPB2, or COPB1 knockdowns were measured at hourly intervals over a 24-h time course, using live cell fluorescence microscopy (Fig. 4E). The resulting time course shows that eGFP expression in COPA, COPB2, and COPB1 knockdown cells mirrors the findings at 24 hpi, with all three knockdowns causing a consistent reduction in eGFP signal throughout the 24-h time period.

Taken together, these findings suggest that COPA, COPB2, and COPB1 knockdown influences a stage of the HAZV replication cycle up to, and including, the translation of viral proteins.

### Localization of COPA in relation to HAZV N protein in infected cells.

**FIG 5:**
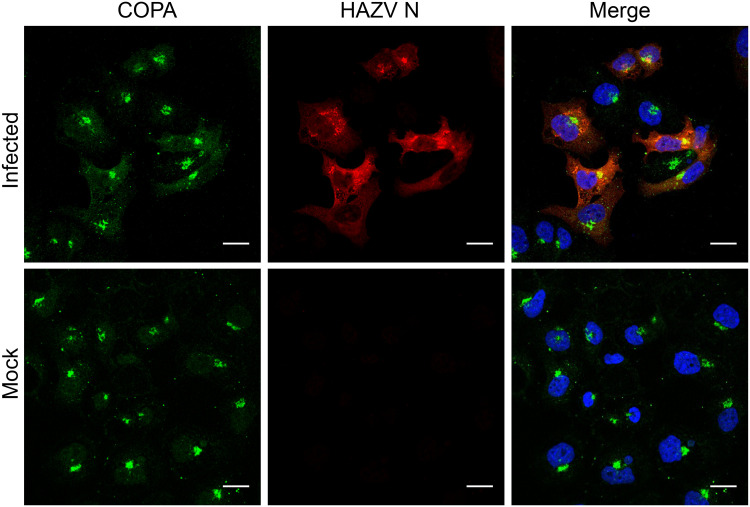
Cellular localization of HAZV N and COPA in SW13 cells. SW13 cells were mock infected or infected at an MOI of 0.25 and imaged at 18 hpi. Scale bar, 10 μm.

To examine whether the influence of COPI complex components was due to direct interaction or sequestration with either a viral protein or virus induced structure, we next examined the spatial location of COPA and the major HAZV structural protein, N. Confocal immunofluorescence microscopy of HAZV-infected SW13 cultures using COPA and HAZV N antisera revealed that COPA staining was predominantly localized within a single large perinuclear structure that exhibited a classical Golgi morphology. In contrast, HAZV N staining appeared in more densely stained perinuclear structures within infected cells, with a secondary diffuse intensity throughout the cytosol. Although regions of HAZV N and COPA staining did overlap in areas, their corresponding peak intensities did not precisely coincide (Fig. 5). Of interest, the distribution of COPA in HAZV-infected cells had noticeably more diffuse cytosolic staining than in uninfected cells within the same field of view (Fig. 5, top left panel), indicating that the distribution of COPA location was influenced by HAZV infection.

### Inhibition of retrograde transport confirms a dependency of HAZV growth on COPI vesicles in an Arf1-independent mechanism.

**FIG 6:**
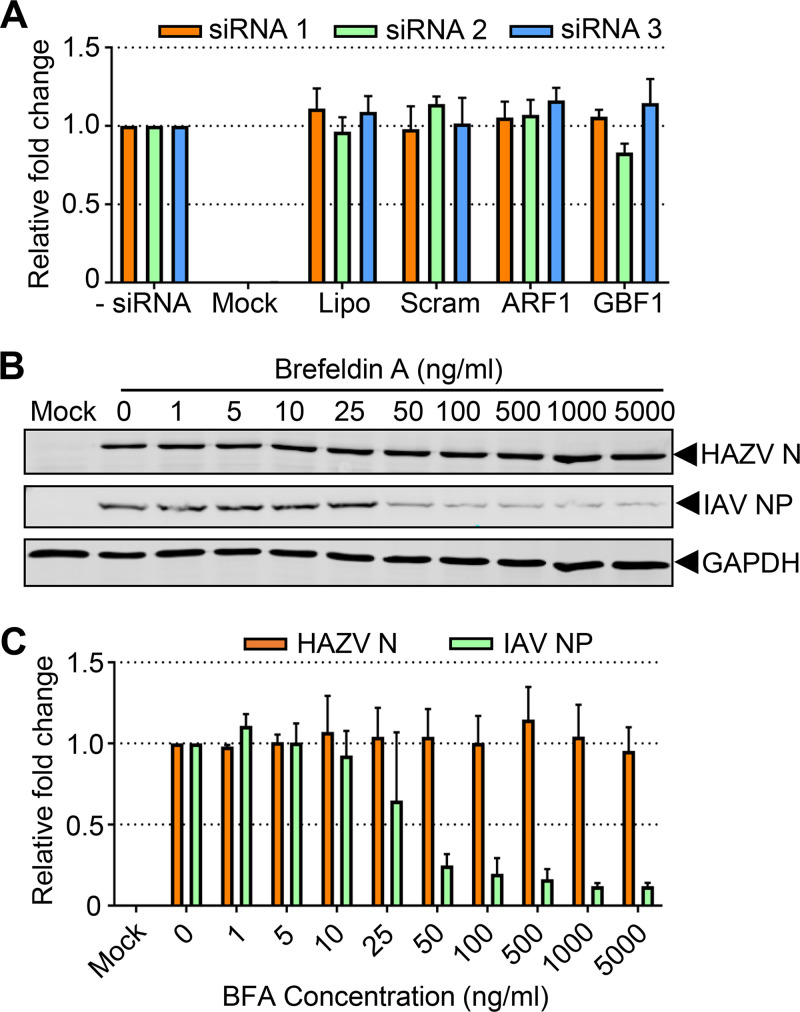
HAZV gene expression in first-round infected cells requires COPA but is Arf1 and GBF1 independent. (A) Histogram of the relative fold change in total integrated intensity of eGFP signal in SW13 cells infected with rHAZV-eGFP at an MOI of 0.25 after independent reverse transfection of triplicate siRNAs (siRNA 1, orange bars; siRNA 2, green bars; siRNA 3, blue bars) against Arf1 and GBF1. Transfection reagent only and scrambled siRNA transfections were included as controls. (B) Western blot analysis of lysates collected from A549 cells pretreated with the indicated concentrations of brefeldin A (BFA) for 45 min prior to infection with rHAZV or rIAV at an MOI of 0.1. Samples were probed with antisera for their respective nucleoproteins (HAZV N or IAV N) and GAPDH as a loading control. Nucleoprotein expression was quantified by densitometric analysis of Western blot data from three independent experimental repeats, with error bars to show variance, represented graphically in panel C.

The formation of COPI vesicles requires the small GTPase ADP-ribosylation factor 1 (Arf1) (36) which, upon activation by guanine exchange factors (GEFs) such as GBF1, becomes GTP bound and anchored within membranes. This GTP-bound form of Arf1 promotes recruitment of the intact heptameric COPI coatomer to its resident membrane (37), three copies of which associate with six copies of Arf1 to form a triad structure, with multiple triads interacting to form COPI vesicles of various sizes. Interestingly, our membrane trafficking siRNA screen suggested no role for either Arf1 or GBF1 in HAZV infection (Fig. 6; see Data Set S1 in the supplemental material), raising the possibility that HAZV requires COPI components for its replication cycle, but in an Arf1- and GBF1-independent manner.

To confirm the Arf1 independence of HAZV intracellular replication, we examined the growth ability of HAZV in the presence of brefeldin A (BFA), a noncompetitive inhibitor of GEF-mediated Arf1 activation with an established role in blocking COPI vesicle formation and retrograde Golgi-ER trafficking (38). After BFA pretreatment, the cells were infected with rHAZV at an MOI of 0.1, and intracellular virus growth was examined at 24 hpi by Western blotting of cell lysates using N protein antisera. Recombinant H1N1 influenza A virus (rIAV) was included in these assays as a positive control due its known dependence on Arf1 for replication, as well as its sensitivity to BFA (39). As expected, Western blot analysis of IAV-infected cell lysates using IAV NP antisera at 24 hpi revealed a dose-dependent reduction in NP expression in response to increasing BFA concentrations. In contrast, quantification of the levels of HAZV N remained unchanged at all BFA concentrations (Fig. 6B and C), confirming that the dependence of HAZV gene expression on COPI components is Arf1 independent.

### COPI components regulate the assembly of infectious HAZV.

**FIG 7:**
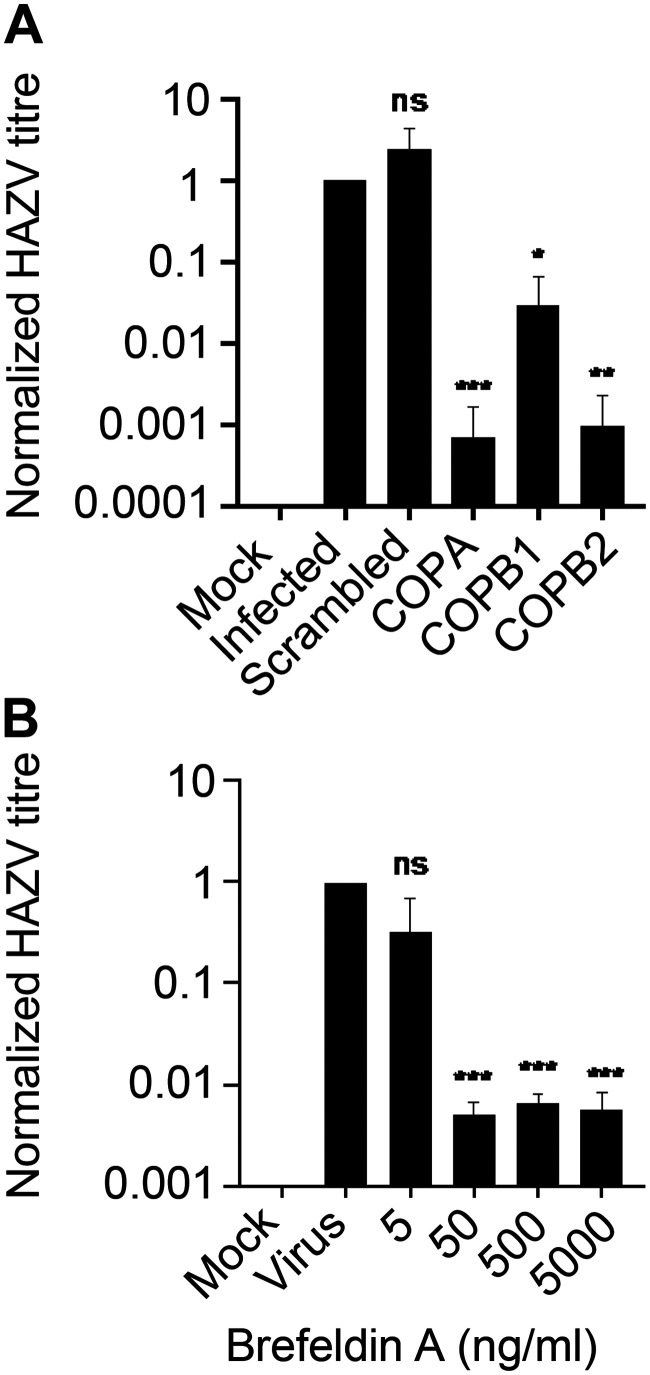
Production of infectious HAZV is reduced by COPA knockdown and brefeldin A treatment. (A) SW13 cell cultures were pretreated with siRNAs targeting COPA, COPB1, and COPB2 and then infected with rHAZV at an MOI of 0.1, alongside cells transfected with scrambled siRNA (Scrambled), cells treated using transfection reagent alone (Infected), and mock-infected cells (Mock) as controls. Supernatants were harvested at 48 hpi, with released virus measured by a plaque assay. (B) Cells were pretreated with brefeldin A at the various stated concentrations and then infected with HAZV at an MOI of 0.1. Released infectious virus in supernatants at 24 hpi was quantified by a plaque assay, measured in duplicate. The statistical significance between conditions is indicated (ns, not significant; *, *P* < 0.1; **, *P* < 0.01; ***, *P* < 0.001).

The quantification of TIIE in cells infected with rHAZV-eGFP at 24 hpi identified an influence of both COPI complex silencing, but not BFA treatment, on the stages of the virus replication cycle up to, and including, protein synthesis. To measure effects of the COPI components on later stages of the HAZV replication cycle, we next analyzed the effects of COPA, COPB2, and COPB1 knockdown on the production of infectious virus. siRNA knockdowns were performed in SW13 cells prior to infection with WT rHAZV at an MOI 0.1; supernatants were then collected at 48 hpi, and titers were calculated by plaque assay. All three siRNA knockdowns resulted in a decrease in virus titers, with the knockdown of COPI cage components COPA and COPB1 in particular, reducing infectious virus production by ∼1,000-fold compared to scrambled siRNA controls (Fig. 7A).

This approximately 1,000-fold reduction in infectious virus production contrasts with the previously measured 2-fold reduction in N production (Fig. 4C and D), or 2- to 5-fold reduction in eGFP expression (Fig. 4E). One possible explanation to account for this discrepancy is that knockdown of COPI complex components exerts an influence at two or more stages of the HAZV replication cycle, with a minor effect at an early stage, prior to, or including virus protein translation, and also with a major effect at a later stage that is required for infectious virus production.

### Brefeldin A-mediated inhibition of retrograde transport reveals that the production of infectious HAZV is Arf1 dependent.

The results described above showed that siRNA knockdown of COPI components influenced HAZV gene expression in an Arf1-independent manner and, in addition, reduced the production of infectious HAZV particles. To extend these studies and to establish whether this COPI dependence was also Arf1-independent, we next investigated whether the production of infectious HAZV was sensitive to BFA treatment.

Following BFA pretreatment at a range of concentrations, SW13 cells were infected with rHAZV at an MOI of 0.1, and at 48 hpi released infectious virus in supernatants was quantified by using a plaque assay (Fig. 7B). BFA treatment markedly reduced the production of infectious HAZV, with a BFA concentration of 50 ng/ml reducing released titers by >100-fold.

Taken together, these results show that the HAZV replication cycle can be divided into at least two distinct phases based on the relative influence of COPI and dependence on Arf1 functionality: an early phase prior to and including protein production that exhibits a minor COPI dependence and is Arf1 independent, followed by a second phase involved in infectious virus production that is highly COPI dependent and dependent on Arf1 function.

## DISCUSSION

Here, we describe the development of rHAZV-eGFP, a modified recombinant HAZV able to express eGFP and its use in the identification of host cell factors involved in nairovirus replication. The multistep growth kinetics of rHAZV-eGFP and WT rHAZV were similar, with an ∼2-fold reduction in rHAZV-eGFP titer at most; this is not surprising given the increased size of the S segment and a possible reduction in expression of N protein downstream of a P2A linker. This virus has great utility, allowing the rapid and simple real-time analysis of nairovirus growth and infectivity, with the added benefit that it requires only low BSL-2 containment, in contrast to other nairoviruses that require a higher containment infrastructure.

The measurement of eGFP expression in rHAZV-eGFP-infected cells provided a convenient and rapid assessment of virus activities using live cell imaging, and these properties were exploited in an siRNA-based screen to identify membrane trafficking factors required during the nairovirus replication cycle. Analysis of viral-eGFP expression in cells treated with a total of 426 siRNAs, specific for 142 target genes, revealed an important role for several components of the COPI coatomer complex, in particular COPA, COPB1, and COPB2. Knockdown of these targets affected eGFP expression and N protein production by a factor of around 2-fold, implying the corresponding COPI targets play a role in virus activities up to and including protein expression. In contrast, knockdown of COPI component expression reduced infectious virus production by a factor of around 1,000.

Since canonical COPI function in vesicle trafficking requires involvement and activation of Arf1 by GEFs (36), we next examined whether treatment of HAZV-infected cells with BFA, a noncompetitive inhibitor of GEF function (38, 40), also influenced HAZV gene expression or infectious HAZV release. We showed BFA treatment had no detectable effect on HAZV gene expression in first-round infected cells, whereas BFA treatment reduced infectious virus production by 100-fold at higher concentrations. Taken together, our results suggest involvement of COPI components within at least two stages of the HAZV replication cycle: a minor and Arf1-independent role within early stages of the replication cycle and a major and Arf1-dependent role during infectious virus assembly.

This is the first report of COPI complex and Arf1 involvement in the replication cycle of a bunyavirus. Recently, Uukuneimi virus, a member of the *Phenuiviridae* family within the *Bunyavirales* order was shown to require the activity of GBF1 (41), a GEF required for Arf1 activation, and thus it is possible that our findings reported here relate to a common functional requirement of COPI vesicle formation for the *Bunyavirales*. Components of the COPI complex have been implicated in the growth of many viruses, with proposed involvement in several distinct life cycle stages, including entry (42–47), gene expression (48–51), virus assembly, and egress (39, 52, 53).

In view of this previous work, it is interesting to speculate at what stage, or stages, of the nairovirus replication cycle the observed COPI dependence might act. COPI complexes are one of three archetypical protein coats, along with COPII and clathrin. Together, they regulate the formation and delivery of vesicles, along with their cargo, to cellular endomembranes, with COPI vesicles performing primary roles in both intra-Golgi and retrograde Golgi-to-ER transport. The Golgi complex is critical for bunyaviruses for many reasons; bunyaviruses modify the Golgi apparatus to establish replication factories, where viral components are synthesized and virus assembly and budding takes place. The Golgi apparatus is also critical for the processing of the viral envelope glycoproteins Gn and Gc (54–58). Thus, the dependence of infectious HAZV production on COPI components is entirely consistent with these activities, and we hypothesize that COPI vesicles are likely required for the transport of Gn and Gc either between Golgi subcompartments or from the Golgi compartment to specialized virus assembly sites. The mechanism of cargo recognition by the COPI coatomer involves interaction between WD-repeat domains on cage components COP-α (COPA) and COP-β′ (COPB2) that directly bind dilysine motifs (KKxx and KxKxx) on cargo (59). Consistent with a critical role of COPI complex components in HAZV replication and egress, there are five such dilysine motifs in the C terminus of Gc.

The dependence of Arf1 for the production of infectious virus is also consistent with its known role; GTP-bound Arf1 becomes membrane associated and initiates vesicle formation by recruiting the COPI coatomer. Thus, the involvement of both COPI and Arf1 in vesicle transport is intrinsically linked, and blockade of Arf1 function by BFA would be expected to influence delivery of Gn and Gc glycoproteins to Golgi body-derived sites of virus assembly, consistent with our observations. It is also possible that the influence of either COPI knockdown or BFA treatment on virus production is less direct; BFA treatment is known to disrupt cellular glycosylation (60), and blocking COPI activity has been shown to disrupt the recycling of cellular glycotransferases (61), which are responsible for glycan processing of newly synthesized glycoproteins within the various Golgi subcompartments. It is reasonable to propose that during a HAZV infection, COPI complex knockdown or BFA treatment and the subsequent impairment of glycotransferase activity would similarly affect HAZV Gn and Gc glycoproteins processing, resulting in reduced infectious virus production.

Our observations that COPI knockdown reduces HAZV-specific gene expression (Fig. 4B to E) implies an involvement at an early stage of the virus replication cycle, prior to or including translation, and what this stage might be is unclear. While COPI vesicles have been associated with entry of several viruses through the endocytic pathway (42–47), there is considerable evidence to suggest this role is likely to be indirect (39, 42), and we cannot yet rule out this possibility. However, one possibility is that COPI knockdown reduces or prevents production of critical cellular factors involved in early entry stages, such as a cell surface receptor, thus slowing or reducing virus entry. Another possibility is that COPI vesicles are involved in the initial recruitment of components required for virus factory formation, where viral gene expression takes place. If factory formation was interrupted, expression of the viral proteins would be expected to be reduced, consistent with our observations.

Our results using both siRNA knockdown of Arf1 and GBF1, as well as Arf1 pharmacological inhibition, revealed the dependence of COPI was Arf1 independent, and this independence is perplexing. To the best of our knowledge, all COPI complex activities thus far described rely on Arf1, on account of its critical role in recruitment of the entire preassembled heptameric COPI coatomer, prior to vesicle formation. Our findings that COPI components are required in an Arf1-independent manner suggests that COPI coatomer components may play additional roles that do not rely on Arf1, and perhaps such roles are unrelated to vesicle formation. It is intriguing to speculate that this noncanonical method of COPI trafficking may represent a newly recognized feature of the mammalian cellular transport system, adding further complexity to our understanding of cargo-mediated transport.

## MATERIALS AND METHODS

### Plasmid design.

Generation of a plasmid expressing HAZV N and eGFP separated by a P2A linker region was achieved via restriction digest and ligation of pMK-RQ-S with pUC57-Kan-eGFP-P2A (Custom purchase; Genewiz). Restriction digests were performed on 1 μg of pMK-RQ-S and pUC57-Kan-eGFP-P2A with EcoRI and NotI (New England Biolabs) for 1 h at 37°C. Digested products were resolved on 1% agarose gels via electrophoresis at 100 V for 45 min in 1× Tris-acetate-EDTA running buffer. DNA bands corresponding to insert and vector were excised from agarose and purified by using a Monarch DNA gel extraction kit (New England Biolabs) according to the manufacturer’s instructions. Ligations were performed using T4 DNA ligase (New England Biolabs) according to the manufacturer’s instructions with successful colonies identified via colony PCR and DNA sequencing (Genewiz) generating pMK-RQ-S-eGFP.

### Recovery of rHAZV-eGFP.

Six-well plates were seeded with 2 × 10^5^ BSR-T7 cells/well, 1 day prior to transfection, in 2 ml of Dulbecco modified Eagle medium (DMEM) supplemented with 2.5% fetal bovine serum (FBS), 100 U/ml penicillin, and 100 μg/ml streptomycin (2.5% DMEM). After 16 to 24 h, the cells were transfected with 1.2 μg of pMK-RQ-S, pMK-RQ-M, and pMK-RQ-L and 0.6 μg of pCAG-T7pol for WT rHAZV recovery, combined with 2.5 μl of Mirus TransIT-LT1 transfection reagent (Mirus Bio) per μg of DNA, in 200 μl of Opti-MEM. For mutant recovery, the WT plasmid was replaced with pMK-RQ-S-eGFP. A control sample, in which transfection of pMK-RQ-L was omitted, was set up alongside each experiment. At 24 hpt, media containing the transfection mix were removed and replaced with fresh 2.5% DMEM. Reinfection of fresh monolayers was carried out in six-well plates seeded with 2 × 10^5^ SW13 cells/well, 1 day prior to infection, in 2 ml of DMEM supplemented with 10% FBS, 100 U/ml penicillin, and 100 μg/ml streptomycin. Cell supernatants from transfected BSR-T7 cells were collected at 120 hpt, and 300 μl was used to infect fresh SW13 cells for 72, 96, or 120 h in a 6-well plate in DMEM with 2.5% FBS. The IncuCyte live cell imaging system (Sartorius) was used to detect total eGFP-positive cells as a marker for virus infection. Infected SW13 cells were imaged hourly for 48 h to permit analysis of total green cell count over time.

### Virus infections.

SW13 monolayers were infected with HAZV at the specified MOI in serum-free DMEM (SFM) at 37°C. After 1 h, the inoculum was removed, and the cells were washed in phosphate-buffered saline (PBS); fresh 2.5% DMEM was then applied for the duration of the infection.

### Viral titration.

Determination of virus titers was achieved through plaque assays. Supernatant was collected at the time titration was required and serially diluted to infect fresh monolayers of SW13 cells in a 6-well plate. After infection, medium containing virus was removed, cells were washed in ice-cold 1× PBS, a 1:1 ratio of 2.5% DMEM to 1.6% methylcellulose was reapplied, and the cells were incubated a further 6 days prior to fixing and staining with crystal violet. Plaques were then counted, and virus titers were determined.

### Virus growth curves.

To assess viral fitness over time, 2 × 10^6^ SW13 cells were seeded into 75-cm^2^ flasks 1 day prior to infection in 8 ml of DMEM supplemented with 10% FBS. After 24 h, virus was used to infect flasks at the specified MOI in 5 ml of SFM for 1 h with shaking, and an aliquot of the infection medium was collected to permit back-titration, ensuring that equal MOI comparisons were made. After infection, medium containing virus was removed, the cells were washed with PBS, and 7 ml of 2.5% DMEM was reapplied, followed by incubation at 37°C for the remainder of the experiment. At 24-h intervals, 200 μl of supernatant was removed and stored at –80°C until all samples had been collected and stored in a similar manner. Following collection at all time points, the samples were analyzed for infectious virus titers as previously described.

### Reverse transfection of siRNA library.

Trypsinized SW13 cells in 2.5% DMEM were counted using a hemocytometer and used to make a cell suspension containing 1 × 10^5^ cells/ml. A master mix was made containing 0.3 μl of Lipofectamine RNAiMAX reagent (Invitrogen) and 16.7 μl of Opti-MEM per well, and 17 μl of this master mix was pipetted into each well of a 96-well plate. A 3- μl volume of working stock siRNA (1 μM) was pipetted into the transfection master mix and mixed, resulting in a final concentration of 3 pmol of siRNA per well. A 100-μl aliquot of the 1 × 10^5^ cell suspension was then applied per well. The cells were incubated with the transfection mix for 24 h at 37°C, and then 60 μl of the medium was removed and replaced with 140 μl of fresh 2.5% DMEM to dilute out any potential toxic effects of the siRNAs or transfection reagent. At 6 h postdilution, the medium was removed, and the cells were washed in PBS prior to infection with rHAZV-eGFP at an MOI of 0.25 in 100 μl of 2.5% DMEM. At 24 hpt, the eGFP fluorescence intensity was determined using the IncuCyte live cell imaging software as a measure of virus gene expression. The total integrated intensity of eGFP (TIIE; green count units [GCU] × μm^2^/image) was first normalized to confluence per well and then analyzed as a percentage of the total green integrated intensity in positive-control wells containing virus, but omitting siRNA and transfection reagent. Normalized values between two technical repeats were averaged and then averaged across two biological repeats (*n* = 4).

### Validation of siRNA knockdown of COPI-specific targets.

To validate the knockdown observed in the siRNA screen, siRNAs specific to COPA, COPB1, and COPB2 were purchased (Ambion) and reverse transfected into SW13 cells. Briefly, 75 pmol of siRNA was mixed with 4.5 μl of Lipofectamine RNAiMAX in 200 μl of Opti-MEM, followed by incubation at room temperature for 20 min. After incubation, the transfection mix was added dropwise to a 6-well plate and overlaid with 2 ml of cell suspension containing 1 × 10^5^ cells/ml in 2.5% DMEM. At 24 hpt, the medium was changed to fresh 2.5% DMEM for 6 h and then infected with rHAZV at an MOI of 0.1. Cell lysates were collected at 24 hpi for analysis via Western blotting or qPCR, and the supernatant was collected for the determination of virus titers.

### Quantitative PCR.

RNA was harvested from cells of interest via TRIzol extraction. Briefly, cells were washed in nuclease-free PBS and then resuspended in TRI Reagent (Invitrogen); chloroform was then added, and the samples were mixed vigorously and incubated at room temperature for 2 min. Samples were centrifuged at 12,000 × *g*, and the resulting aqueous phase was collected and added to isopropanol to precipitate RNA for 5 min at room temperature. Samples were spun at 12,000 × *g*, and the resulting pellet was washed in ice-cold 75% ethanol prior to resuspension in nuclease-free H_2_O. qPCR was carried out using a One Step Mesa Green qRT-PCR MasterMix for SYBR assay (Eurogentec) according to the manufacturer’s instructions, with samples normalized to GAPDH expression. The primer sequences used were as follows: HAZV S segment (5′-CAA GGC AAG CAT TGC CAC AC-3′ and 5′-GCT TTC TCT CAC CCC TTT TAG GA-3′), COPA (5′-CCA CTA TCA GAA TGC CCT ATA CC-3′ and 5′-CCA CAA ACC CAT CTT CAT CC-3′), COPB1 (5′-ACA GAGA GAA AGA GGC AGC AGA-3′ and 5′-GCA AGG TATA CAC TGG TTT GGT TC-3′), COPB2 (5′-GTG GGG ACA AGC CAT ACC TC 3′ and 5′-GTG CTC TCA AGC CGG TAG G-3′), and GAPDH (5′-TGT GGT CAT GAG TCC TTC CAC GAT-3′ and 5′-AGG GTC ATC ATC TCT GCC CCC TC-3′).

### Immunofluorescence.

Trypsinized SW13 cells were seeded onto 16-mm round glass coverslips (VWR) in a 12-well plate at 5 × 10^4^ cells/well, followed by incubation at 37°C. After 16 to 24 h, the cells were infected at an MOI of 0.1 in SFM for 1 h at 37°C. After the infection medium was removed, the cells were washed in PBS, 2.5% DMEM was applied, and the samples were incubated at 37°C for a further 24 h. Medium was then removed, and the cells were washed twice in PBS prior to fixation in 4% (vol/vol) paraformaldehyde in PBS for 10 min at room temperature. After fixation, the cells were washed twice in PBS and then incubated in permeabilization buffer (0.1% [vol/vol] Triton X-100, 1% [wt/vol] bovine serum albumin [BSA] in 1× PBS) for 15 min at room temperature. The cells were then blocked in blocking buffer (1% [wt/vol] BSA in 1× PBS) for 45 min then incubated with HAZV N antisera (1:1,000) or COPA (1:200) primary antibody for 1 h at room temperature. The cells were washed three times in PBS and then incubated with the corresponding secondary Alexa Fluor antibody (Life Technologies; 1:500 in blocking buffer) for 1 h at room temperature in a light protected vessel, followed by three washes with PBS. Coverslips were then mounted onto glass slides with the addition of ProLong Gold Antifade reagent with DAPI (Thermo Fisher Scientific), sealed, and stored at 4°C. Images were then taken on an LSM 700 confocal microscope and processed using Zen (Blue Edition) software.

### Inhibition of retrograde transport.

Trypsinized A549 cells were seeded into 12-well plates at 1 × 10^5^ cells/well and incubated at 37°C. After 16 to 24 h, the cells were pretreated with BFA at the indicated concentrations for 45 min in SFM prior to infection with rHAZV or rIAV at an MOI of 0.1 for 1 h at 37°C. After the infection period, medium containing virus was removed, and cell monolayers were washed three times with PBS. Fresh 2.5% DMEM was then reapplied containing the indicated concentration of BFA for a further 24 h. At this point, lysates were collected and analyzed via Western blotting, and supernatant was collected for the determination of virus titers.

### Statistical analyses.

Statistical analyses were performed using an unpaired *t* test to determine statistically significant differences between treatments (ns, nonsignificant; *, *P* < 0.1; **, *P* < 0.01; ***, *P* < 0.001).

## Supplementary Material

Supplemental file 1
